# Herbal Extract from *Codonopsis pilosula* (Franch.) Nannf. Enhances Cardiogenic Differentiation and Improves the Function of Infarcted Rat Hearts

**DOI:** 10.3390/life11050422

**Published:** 2021-05-05

**Authors:** Jieh-Neng Wang, Chung-Dann Kan, Lain-Tze Lee, Lynn L. H. Huang, Ya-Li Hsiao, Allen H. Chang, Wanchun Liu, Cheng Lin, Chou-Wen Lin

**Affiliations:** 1 Department of Pediatrics, National Cheng Kung University Hospital, College of Medicine, National Cheng Kung University, Tainan 70401, Taiwan; 2 Department of Surgery, National Cheng Kung University Hospital, College of Medicine, National Cheng Kung University, Tainan 70401, Taiwan; bsmlily@gmail.com; 3 Herbal Medical Product Technology Division, Biomedical Technology and Device Research Laboratories, Industrial Technology Research Institute, Hsinchu 31057, Taiwan; lens@gp-corp.com (L.-T.L.); wanchunliu@itri.org.tw (W.L.); chenglin@gp-corp.com (C.L.); 4 Department of Biotechnology and Bioindustry Sciences, National Cheng Kung University, Tainan 70401, Taiwan; lynn@mail.ncku.edu.tw (L.L.H.H.); sparktrix@gmail.com (A.H.C.); 5 Gold Medal Glycine Tomentella Hayata Biological Technology Company, Tainan 73052, Taiwan

**Keywords:** herbal extracts, cardiogenesis, cardiomyocytes, myocardial infarction

## Abstract

Background: The roots of *Codonopsis pilosula* (Franch.) Nannf. have been used in traditional Chinese medicine for treating cardiovascular disease. In the current study, we aimed to discover herbal extracts from *C. pilosula* that are capable of improving cardiac function of infarcted hearts to develop a potential therapeutic approach. Methods: A mouse embryonic stem (ES) cell-based model with an enhanced green fluorescent protein (eGFP) reporter driven by a cardiomyocyte-specific promoter, the α-myosin heavy chain, was constructed to evaluate the cardiogenic activity of herbal extracts. Then, herbal extracts from *C. pilosula* with cardiogenic activity based on an increase in eGFP expression during ES cell differentiation were further tested in a rat myocardial infarction model with left anterior descending artery (LAD) ligation. Cardiac function assessments were performed using echocardiography, 1, 3, and 6 weeks post LAD ligation. Results: The herbal extract 417W from *C. pilosula* was capable of enhancing cardiogenic differentiation in mouse ES cells in vitro. Echocardiography results in the LAD-ligated rat model revealed significant improvements in the infarcted hearts at least 6 weeks after 417W treatment that were determined based on left ventricle fractional shortening (FS), fractional area contraction (FAC), and ejection fraction (EF). Conclusions: The herbal extract 417W can enhance the cardiogenic differentiation of ES cells and improve the cardiac function of infarcted hearts.

## 1. Introduction

Heart failure (HF) is a frequent complication of myocardial infarction (MI) [1]. Many acute and chronic adaptations occur due to MI that progresses to HF, for example, neurohumoral hyperactivity, oxidative stress, inflammatory response, and cardiac remodeling [2]. According to pathophysiology, myocardial ischemia leads to ultrastructural damage, contractility dysfunction, an increase in reactive oxygen species (ROS) production, and changes in myocardial metabolism which culminate in necrosis of cells [3]. Mitochondrial dysfunction is a hallmark of heart failure, and a marker of both oxidative stress and inflammation. Myocardial injury leads to activation of a stereotyped inflammatory cascade comprised of early neutrophil ingress followed by monocyte-macrophage infiltration [4]. Recent studies have revealed oxidative stress and inflammation to be key pathophysiological elements of heart failure syndrome. Oxidative stress and inflammation are closely connected to each other, both in the acute phase after myocardial infarction and during chronic cardiac remodeling [5]. The precise contribution of the different pathophysiological components (e.g., microvascular dysfunction and inflammation) to injury is likely to be heterogeneous, and therefore understanding mechanistic pathways in specific patient subgroups is key to identifying novel therapeutic strategies [6].

However, current standard treatments for HF, including pharmacological and surgical interventions, have their limitations. These therapeutic interventions cannot restore damaged heart tissues and are always associated with side effects [7]. As such, alternative treatments that can restore injured myocardium have been the current focus of HF intervention. Among the emerging alternative strategies for treating HF, stem cells (including ES cells), induced pluripotent stem (iPS) cells, and multipotent adult stem cells have shown promise [8,9,10]. These stem cells can be coaxed in vitro into cardiac progenitors which, then, differentiate into cardiomyocytes that replace infarcted cardiac tissues and eventually restore normal heart function. However, the potential risk of neoplastic transformation from the transplantation of unguided non-cardiac progenitors has remained a concern [11]. Other challenges to this type of stem cell therapy include expansion of appropriate stem cell populations, restricted homing, loss of the majority (95%) of infused stem cells, functional integration of transplanted stem cells, and delivery strategies [7].

Another therapeutic strategy for HF treatment has been to initiate a cardiogenic activity in vivo by promoting myocardial differentiation of circulated stem cells or by activating the proliferation and differentiation of resident cardiac stem or progenitor cells to regenerate new heart tissues. This strategy requires no invasive therapeutic interventions for infarcted myocardium treatment. Although the fundamental mechanism underlying cardiogenesis has not been fully elucidated, some laboratories have searched for molecules with cardiogenic activity that may serve therapeutic purposes in patients with MI [12,13,14,15].

While there is a strong conceptual framework for antioxidant or anti-inflammatory strategies as adjunctive therapies for the treatment of heart failure, these drugs have only been evaluated in a few clinical trials [5]. Recent studies have shown that Chinese herbal medicines, widely utilized for thousands of years in Asian countries, possess anti-inflammatory and antioxidant functions that can help with cardiovascular diseases [16,17,18]. *Codonopsis pilosula* is a perennial flowering plant species native to Northeast Asia and Korea that usually grows around stream banks and forest openings under the shade of trees. The roots of *C. pilosula* (radix) have been used in traditional Chinese medicine to lower blood pressure, increase red and white blood cell counts, cure appetite loss, strengthen the immune system, and replenish chi [19]. Moreover, studies have shown some potential benefits for patients with AMI, such as reduced cardiac death and HF, although the precise mechanisms have remained unclear [20,21,22]. Therefore, we hypothesized that the herbal extract derived from the water-solubilized fraction of *Codonopsis pilosula* (Franch.) Nannf. could contain cardiogenic activity and improve cardiac function of infarcted rat hearts.

## 2. Materials and Methods

### 2.1. Vector

The mouse α-myosin heavy chain (α-MHC) promoter was generously provided by Jeffrey Robbins [23], while the DNA fragment of the BamHI-SalI α-MHC promoter (5.5-kb fragment) was inserted into the BglII-SalI site of the multiple cloning site of pEGFP-1 (Clontech, Palo Alto, CA, USA) to form pMGN22 for the transfection of ES cells.

### 2.2. Embryonic Stem Cell Lines

A transgenic ES cell line was created by transfecting pMGN22 into mouse ES-D3 cells (ATCC) using Lipofectamine 2000 (Invitrogen, Carlsbad, CA, USA), as described by the manufacturer. Stable transgenic ES cells, EMG8, were selected in the presence of 500 μg/mL G418 (Sigma, St. Louis, MO, USA) on a feeder cell-free culture plate coated with 0.1% gelatin (Millipore, Billerica, MA, USA) in ES medium containing Dulbecco’s Modified Eagle’s Medium (Gibco, Paisley, Scotland, UK), 0.1 mM non-essential amino acids (Gibco), 0.15 mM α-monothioglycerol (ICN Biomedicals Inc., Costa Mesa, CA, USA), 15% ES cell-qualified fetal bovine serum (Gibco), penicillin G (100 U/mL), 100 μg/mL streptomycin, 250 ng/mL amphotericin B (Sigma), and 10^3^ U/mL ESGRO (Chemicon, Temecula, CA, USA). Then, EMG8 cells were cultured in ES medium containing 250 μg/mL G418 in a 5% CO_2_ environment at 37 °C, and the medium was changed every other day.

### 2.3. Embryonic Stem Cell Differentiation

To initiate spontaneous differentiation of mouse ES cells, EMG8 cells were seeded at a density of 2000 cells/well in a 96-well plate (Corning, Corning, New York, NY, USA) coated with 0.1% gelatin (Millipore) in ES differentiation medium containing high-glucose Dulbecco’s Modified Eagles’s Medium (Gibco), 0.1 mM non-essential amino acids (Gibco), 0.1 mM β-mercaptoethanol (Sigma), 20% fetal bovine serum (Gibco), 100 U/mL penicillin G (Gibco), 100 μg/mL streptomycin (Gibco), 250 ng/mL amphotericin B (Sigma), and 250 μg/mL G418 (Sigma). The medium was replaced every other day, and the enhanced green fluorescent protein (eGFP) intensity was measured after 10 days using a SpectraMax M2 microplate reader (Molecular Devices, Sunnyvale, CA, USA) at excitation and emission wavelengths of 488 and 519 nm, respectively. However, spontaneous differentiation of EMG8 cells were also conducted through the formation of embryoid bodies (EBs) [24,25]. Briefly, the EB was formed in a hanging drop containing 500 cells in 25 μL of ES differentiation medium on the cover of a petri dish (SPL) and cultured in a cell culture incubator with 5% CO_2_ at 37 °C for two days, followed by resuspension onto petri dishes in ES differentiation medium for another 5 days of growth. Then, EBs were transferred to gelatin-coated 6-well plates (Nunc, Roskilde, Denmark ) in ES differentiation medium for continuous differentiation to examine contractile cell foci and eGFP fluorescence. Images of the contractile EB outgrowths expressing eGFP were taken using a Leica DM IRBE microscope (Leica Microsystems, Heidelberg, Germany).

### 2.4. Preparation of Herbal Extracts

The herb used in this study was acquired from a farm in Hubei province, China. A total of 100 g of the herb powder was extracted in 1 L of water at 95 °C using the hot backflow extraction method for 2.5 h. After cooling the crude extract, the clear extract was separated from the crude extract either by a Whatman No.1 filter (Whatman International, Maidstone, UK) with suction or by centrifugation, and then concentrated, frozen, and lyophilized to create a dry powder for long-term storage. To evaluate cardiogenic activity, the herbal extract was re-dissolved in ddH_2_O to a concentration of 100 μg/mL.

### 2.5. Immunocytochemical Staining

EMG8-derived EB outgrowths seeded on gelatin-coated glass slides after 15 days of differentiation were fixed with 4% formaldehyde at room temperature for 20 min, permeabilized with 0.5% Triton X-100 (Sigma) for 5 min, and then blocked with 5% normal goat serum (NGS) in PBS at room temperature for 20 min. Then, EB outgrowths were incubated with primary antibody (rabbit anti-Nkx2.5,1:150 dilution, GeneTex, Irvine, CA, USA) or mouse anti-α-actinin IgG1 (1:200 dilution, Enzo, Farmingdale, NY, USA) in 1% NGS/PBS at 4 °C overnight. After washing twice with PBS, EB outgrowths were exposed to secondary antibodies ((goat-anti-rabbit Qdot655-conjugated IgG, 1:200 dilution, Invitrogen, Carlsbad, CA, USA) or goat-anti-mouse TRICT-conjugated IgG (1:200 dilution, Jackson ImmunoResearch, West Grove, PA, USA)) at room temperature for 1 h. Then, images of immunostained cells were taken using a Leica DM IRBE microscope (Leica Microsystems, Heidelberg, Germany).

### 2.6. Effects of Herbal Extracts on Differentiating EMG8 Cells

To test which herbs had the potential cardiogenic activity, more than 100 herbal extracts were prepared from our database (data not shown). Here, only six herbal extracts (0041W, *Bupleurum chinense*; 148W, *Lobelia chinensis*; 154W, *Lophatherum gracile*; 165W, *Lithospermum erythrorhizon*; 239W, *Forsythia suspense*; and 417W, *Codonopsis pilosula* (Franch.)) are shown as the representatives from many other tests. In addition, ascorbic acid (Vitamin C), which was reported to enhance differentiation of ES cells into cardiomyocytes [14], was used as a positive control. To determine the cardiogenic activity of herbal extracts, undifferentiated EMG8 cells were seeded onto a gelatin-coated 96-well plate at 2000 cells/well in ES differentiation medium. Herbal extracts were added to each well on Day 2, with five replicates for each sample, at a concentration of 0.5 mg/ml. On Day 11, the medium on each well was removed, and the fluorescence was analyzed in a microplate reader at excitation and emission wavelengths of 488 and 519 nm, respectively. Undifferentiated EMG8 cells were subjected to spontaneous differentiation in the presence of different herb extract dosing were also tested. The eGFP fluorescence intensities were measured on Day 9 and were presented as the mean value of five replicates.

### 2.7. Experimental Animals

Male inbred Wistar rats (3 months old, weighing 250–300 grams, Animal Center of the National Cheng Kung University Medical College) were used as the experimental animals. The were six rats in the control group, and the same number of rats was in the test group. All animal procedures were carried out with the approval of the Animal Care Committee of the National Cheng Kung University Hospital Research Institute (IACUC approval no. 102179) and in compliance with the *Guide for the Care and Use of Laboratory Animals* by the Institute of Laboratory Animal Resource. Briefly, all rats were housed in cages at room temperature (20–26 °C) in an atmosphere with 60–70% humidity and under a 12/12 h light/dark cycle with unrestricted access to water and food.

### 2.8. The Rat Myocardial Infarction Model

After the induction of anesthesia through isoflurane inhalation in an anesthetic chamber, rats were intubated with a 16-G angiocatheter and ventilated with positive-pressure ventilation using a Harvard ventilator. Anesthesia was maintained with 2% inhaled isoflurane (Panion & Bf Biotech, Taoyuan, Taiwan), and electrocardiograph (EKG) monitoring was established. A left anterior thoracotomy was performed, after which the heart was visualized from the fourth intercostal space through a pericardial incision. Then, the left anterior descending artery (LAD) was encircled and ligated with a 7-0 proline stitch, as previously reported [26]. Myocardial ischemia was confirmed through visual inspection and EKG ST-segment elevation during the operation. The ribs and subcutaneous and skin incisions were closed with 3-0 Vicryl (Ethicon Co, Inc., Sommerville, NJ, USA) and 3-0 Ethilon (Ethicon Co, Inc) in layered sutures. Rats received identical amounts of intramuscular analgesia (meperidine, 0.3–0.5 mg/100 g body weight) for pain control after surgery.

### 2.9. Effects of Herbal Extracts in the Rat Myocardial Infarction Model and Cardiac Function Assessment

Further tests for the cardiogenic effect of herbal extracts were conducted in an in vivo animal model. One day after LAD ligation, the rats were divided into the following two groups: the control group (*n* = 6) which received normal saline, and the test group (*n* = 6) which received 10 mg of herbal extracts/rat dissolved in 100 μL of distilled water daily for 10 consecutive days by intraperitoneal injection (IP). After 1, 3, and 6 weeks, cardiac function and affected wall size were measured using echocardiography to collect data. Rats were anesthetized and placed in the left-lateral decubitus position. Short-axis two-dimensional images at the mid-papillary level of the left ventricle (LV) were stored as digital loops, and both the end-systolic (ESA) and end-diastolic (EDA) cavity areas were determined by tracing the endocardial borders. The fractiona shortening (FS) was calculated from M-mode images as ((LVEDD − LVESD)/LVEDD) × 100, where LVEDD represents the LV end-diastolic dimension and LVESD represents the LV end-systolic dimension. The FAC was calculated as ((EDA − ESA)/EDA) × 100, while the EF was calculated as (SV/EDV) × 100, where SV is the stroke volume and is equal to end-diastolic volume (EDV) minus the end-systolic volume.

**Figure 1:**
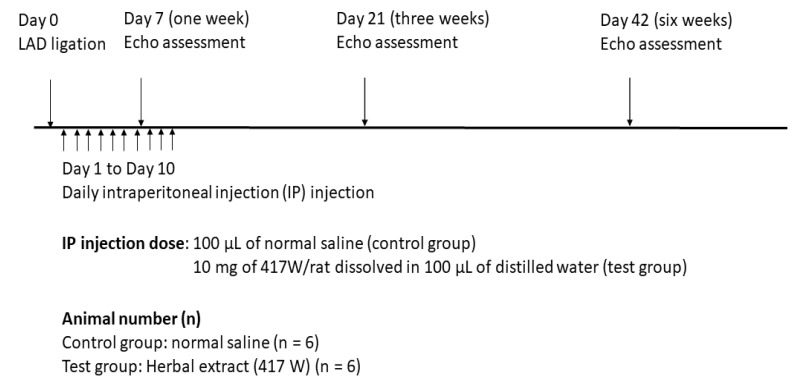
Animal study protocol.

Measurements were taken three times, the average of which was used for analysis. After positive LAD ligation determined through echocardiography according to the random digits tables, rats were randomized into two study groups, i.e., the control group and the herbal extracts treatment group. Thereafter, an observer blinded to the study groups repeated the cardiac function assessment using echocardiography. The animal study protocol is shown below (Figure 1).

## 3. Results

### 3.1. Cardiomyocyte Identity of Enhanced Green Fluorescent Protein (eGFP)-Expressing EB Outgrowths

To establish the ES cell-based model for evaluating herbal extracts with cardiogenic activity, transgenic ES cells (EMG8) were created to express eGFP fluorescence under a cardiac-specific α-MHC promoter. Given that α-MHC is a cardiomyocyte-specific gene expressed early in the developing heart [27], eGFP expression would be associated with cardiomyocytes in differentiating ES cells.

**Figure 2:**
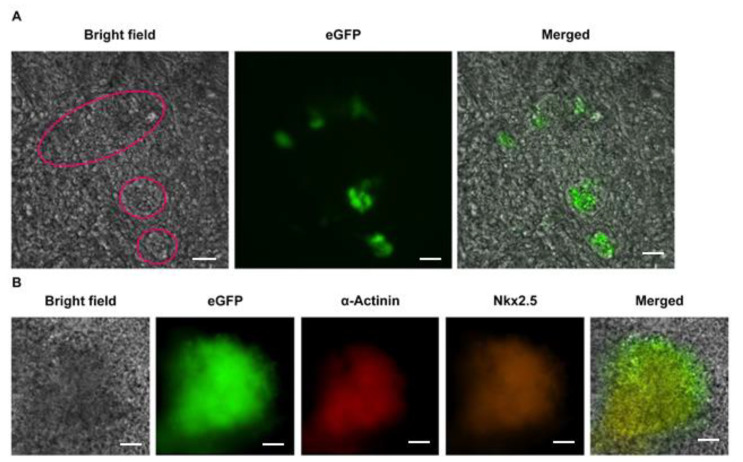
Cardiomyocyte identity of enhanced green fluorescent protein (eGFP)-expressing contractile cardiogenic foci. (**A**) Contractile cardiogenic foci within the red circles overlaid with eGFP-expressing cells; (**B**) these EMG8-derived eGFP-expressing contractile cardiogenic foci within EB outgrowths were stained with antibodies against both cardiomyocyte-specific α-actinin and Nkx2.5. Bar = 50 µm in A, bar = 200 µm in B.

EMG8 cells were subjected to EB formation through the hanging drop method. eGFP expression within EB outgrowths on Day 15 was examined under an epifluorescence microscope. The bright-field image on the left panel of Figure 2A shows the contractile cell foci within the circular area. The green fluorescence of eGFP expression was also detected on the EB outgrowths (middle) and exclusively overlaid with those contractile cardiogenic foci (right panel), suggesting that these contractile cells expressed eGFP.

To further confirm the identity of eGFP-expressing contractile cardiogenic foci, EB outgrowths were subjected to immunocytochemistry. The results presented in Figure 2B show that these eGFP-expressing contractile cells were stained with antibodies against both cardiomyocyte-specific proteins, i.e., α-actinin and Nkx2.5, further proving that these contractile cells were cardiomyocytes.

### 3.2. eGFP Expression on Spontaneous Differentiating EMG8 Cells

**Figure 3:**
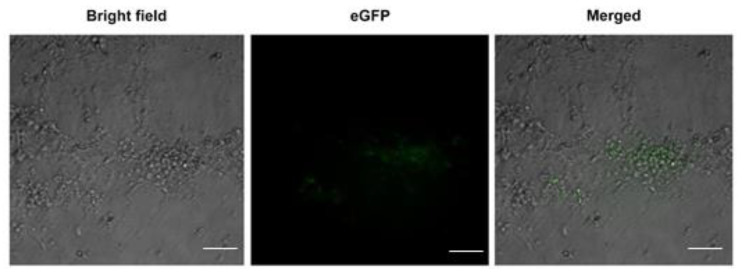
eGFP-expressing cardiomyocytes can be observed among spontaneously differentiating EMG8 cells without EB formation. Bar = 100 µm.

To use the spontaneous differentiation of EMG8 cells directly, without EB formation, as the cell-based model for evaluating cardiogenic activity of herbal extracts, in this study, we also examined whether EMG8 cells could undergo cardiogenic differentiation without the EB formation stage. To this end, EMG8 cells were seeded directly onto a culture plate in the presence of ES differentiation medium. After 10 days, differentiating EMG8 cells were subjected to epifluorescence microscopy to examine eGFP fluorescence expression. Figure 3 shows the presence of eGFP-positive cells, suggesting that cardiomyocyte formation could also occur through spontaneous differentiation of EMG8 cells without EB formation. Hence, an evaluation of herb extracts with cardiogenic activity could be performed by directly seeding EMG8 cells onto 96-well plates in the presence of herbal extracts.

### 3.3. Resul ts of Herbal Extracts on Differentiating EMG8 Cells

**Figure 4:**
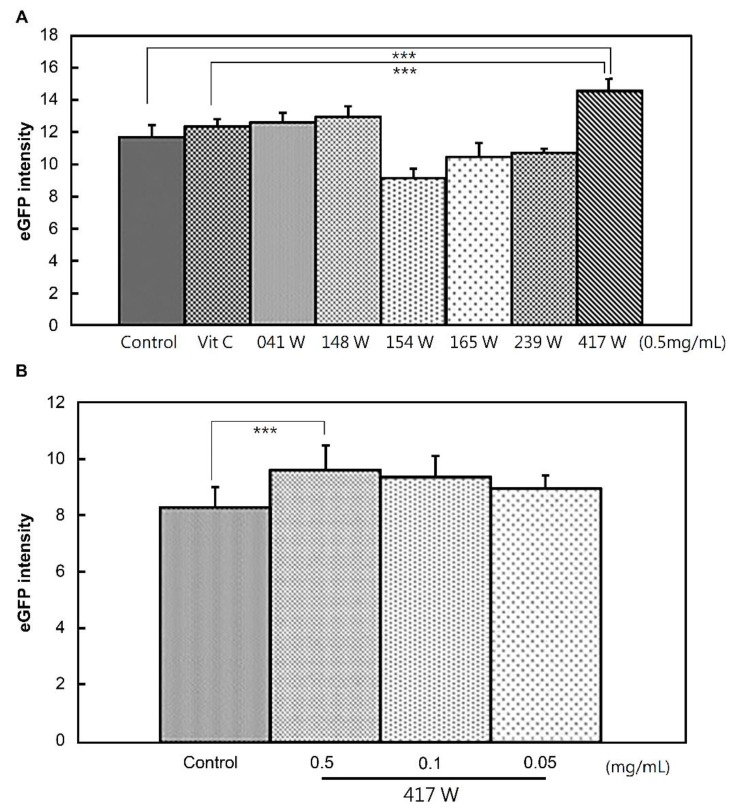
Herbal extract 417 W exhibited potential cardiogenic activity. (**A**) Different herbal extracts were added to undifferentiated EMG8 cells at 0.5 mg/mL. Significant differences were shown using a one-way ANOVA test. Post hoc two-by-two comparison using Fisher’s least significant difference (LSD) demonstrated a difference between 417 W and vitamin C (*p* < 0.001) and control (*p* < 0.001) as compared with the other herbal extracts; (**B**) undifferentiated EMG8 cells were subjected to spontaneous differentiation in the presence of 417 W at 0.5, 0.1, and 0.05 mg/mL. Student t-test showed that e-GFP intensity was significantly higher in the 417 W (0.5 mg/mL) group than in the control group *** *p* < 0.001.

Among the herbal extracts tested, extract 417W, which indicated the water-solubilized extract of *Codonopsis pilosula* (Franch.) Nannf., showed significant cardiogenic activity that was 24.2% better than the herbal extracts and the control set, as shown in Figure 4A. The 417W extract also showed better cardiogenic activity than ascorbic acid (vitamin C). Moreover, the cardiogenic differentiation of 417W into EMG8 cells was dose dependent, as shown in Figure 4B.

The cardiogenic activity of 417W was also examined using the model of EB formation. Accordingly, 417W promoted more beating EB outgrowths as compared with other herbal extracts, which was consistent with observations using the model of ES cell differentiation without EB formation (data not shown). The aforementioned results suggested that 417W could significantly enhance cardiogenic differentiation of undifferentiated EMG8 cells.

### 3.4. Results of 417W in the Rat Myocardial Infarction Model

After 1, 3, and 6 weeks, FS, FAC, and EF of the treated hearts were assessed, with the corresponding values being summarized in Table 1.

**Figure 5:**
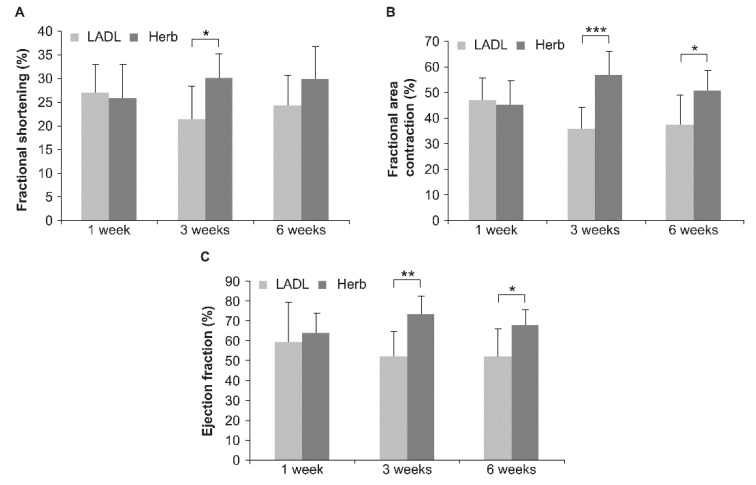
417W treatment improved cardiac function in left anterior descending artery (LAD)-ligated rats. The FS, FAC, and EF were monitored 1, 3, and 6 weeks post LAD ligation (**A**, **B**, and **C**, respectively). * *p* < 0.05, ** *p* < 0.005, and *** *p* < 0.001 as compared with the control group.

The echocardiography results, shown in Figure 5, revealed no significant improvement 1 week after 417W treatment. However, 3 weeks after treatment, 417W-treated rats (*n* = 6) showed a 40.8% improvement in FS (*p* < 0.05), 59.1% improvement in FAC (*p* < 0.001), and 40.2% improvement in EF (*p* < 0.005) as compared with the control group of LAD-ligated rats (*n* = 6). After 6 weeks, a 22.6% improvement in FS, 35.3% (*p* < 0.05) improvement in FAC, and 29.6% (*p* < 0.05) improvement in EF were noted. Although both FAC and EF in the 417W-treated group declined from week three to week six post 417W treatment, the functional improvement in the infarcted hearts was still considerably more significant than in the control group, thereby confirming that herbal extract 417W could enhance the function of infarcted hearts.

## 4. Discussion

The current study tested the cardiogenic activity of water-solubilized herbal extracts using a transgenic ES cell-based model. Our in vitro tests found that the water-solubilized extract, 417W from *Codonopsis pilosula* (Franch.) Nannf., showed cardiogenic activity, suggesting it has potential for promoting cardiac differentiation of ES cells in vitro. Consistent with this result, the infarcted hearts of the LAD-ligated rat model treated with 417W also showed functional improvements in FS, FAC, and EF, although whether these improvements were due to the replenishment of cardiomyocytes derived from stem cells in the circulation, resident cardiac stem cells, or other mechanisms requires further investigation.

The molecular pathways underlying heart formation are complicated and require further exploration. Cardiac differentiation in the embryo originates from the anterolateral mesoderm that receives instructive guidance from the adjacent endoderm [28,29]. Both bone morphogenetic protein and fibroblast growth factor families of signaling molecules expressed in the endoderm of the cardiogenic region are involved in the determination of cardiac fate [30,31], while Nkx2.5, MEF-C, and the GATA family are responsible for initiating cardiomyocyte differentiation [32,33,34]. Moreover, evidence has shown that non-canonical Wnt/JNK signaling molecules can promote cardiogenesis during embryogenesis and also in adult stem cells [35,36]. Hence, granted that 417W acts on the cardiogenic program, these factors could possibly account for its cardiogenic activity.

Over the years, laboratory data have shown that many medicinal herbs, the most famous being *Ginseng*, *Ginkgo biloba*, *Ganoderma lucidum*, and *Gynostemma pentaphyllum*, may have therapeutic value in cardiovascular diseases (CVD) as they can interfere with several CVD risk factors [37]. Ginseng and its ginsenoside constituents have vasorelaxation, antioxidation, anti-inflammation, and anticancer activities [38]. *Codonopsis pilosula* (Franch.) Nannf., the root of the Codonopsis genus, has been used for the treatment of various diseases. Other studies reported that *Codonopsis pilosula* (Franch.) Nannf. regulated gastric basic electrical rhythm disorder under stress conditions and inhibited gastric motility in rats with acute gastric ulcer [39]. It has also been used to revitalize spleen and liver function [40]. Total alkaloids extracted from *Codonopsis pilosula* (Franch.) Nannf. with 20% alcohol were able to enhance neurite outgrowth induced by nerve growth factor in PC12 cells [41], indicating that different components of *Codonopsis pilosula* (Franch.) Nannf. may be responsible for different therapeutic effects. Because the herbal extract protocols are not the same, this 417W extract contains many ingredients and has not been studied completely yet. As more studies are being continued, new components from the 417W extract have been discovered, such as saponin, alkaloids, atrctylenolide III, angelicin, and psoralen. Therefore, currently, it is hard to know exactly what possible components 417W extract contains, and which component in 417W extract plays the major role on enhancing cardiogenic differentiation. Therefore, the mechanism through which *Codonopsis pilosula* (Franch.) Nannf. exerts its cardiovascular therapeutic effects still remains unclear. Notably, Tsai et al. [22] found that *Codonopsis pilosula* (Franch.) Nannf. attenuated the cardiac-impaired insulin-like growth factor (IGF) II receptor pathway in myocardial cells, while Chang et al. [20] reported that it suppressed the apoptotic pathway enhanced by AngII plus Leu27-IGFII in myocardial cells. Therefore, the active components of 417W responsible for cardiogenic activity require further purification and examination before any applications.

## 5. Conclusions

Our study demonstrated that the herbal extract 417W from the water-solubilized fraction of *Codonopsis pilosula* (Franch.) Nannf. exhibited cardiogenic activity in both the ES cell-based model and the in vivo LAD-ligated rat model. Hence, this screening system can be a useful method for evaluating herbal extracts with cardiogenic activity; however, further studies are needed.

## Figures and Tables

**Table 1:** Fractional shortening (FS), frctional area contraction (FAC), and ejection fraction (EF) values collected from the control and 417W-treated mice 1, 3, and 6 weeks post left anterior descending artery ligation. Data are expressed as the mean (%) ± standard deviation of six replicates.

	Control (*n* = 6)			417W (*n* = 6)		
	1 week	3 weeks	6 weeks	1 week	3 weeks	6 weeks
FS (%)	26.9 ± 6.0	21.3 ± 7.1	24.3 ± 6.4	25.8 ± 7.1	30.0 ± 5.2	29.8 ± 6.9
FAC (%)	46.9 ± 8.7	35.7 ± 8.5	37.4 ± 11.3	45.2 ± 9.3	56.8 ± 9.0	50.6 ± 8.0
EF (%)	59.4 ± 19.9	52.2 ± 12.4	52.1 ± 13.6	56.8 ± 9.8	73.2 ± 9.2	67.5 ± 7.9

FS, fractional shortening; FAC, fractional area contraction; EF, ejection fraction.

## Data Availability

Data available on request due to institute restrictions.
